# Poor prognosis of acute kidney injury superimposed on chronic kidney disease: potential mechanisms, risk factors and novel biomarkers

**DOI:** 10.3389/fmolb.2026.1879518

**Published:** 2026-09-03

**Authors:** Linmei Gao, Yuming Ding, Yanru Zhao, Yi Chen, Tao Liu, Wentong Lin, Bo Yang

**Affiliations:** 1 Department of Nephrology, First Teaching Hospital of Tianjin University of Traditional Chinese Medicine, Tianjin, China; 2 National Clinical Research Center for Chinese Medicine, Tianjin, China; 3 Tianjin University of Traditional Chinese Medicine, Tianjin, China

**Keywords:** acute-on-chronic kidney injury, biomarkers, cell cycle arrest, chronic inflammation, maladaptive repair, mitochondrial dysfunction

## Abstract

Acute kidney injury superimposed on chronic kidney disease (acute-on-chronic kidney injury, hereinafter referred to as AoCKI) is a common clinical syndrome with a generally poor prognosis compared to isolated acute kidney injury (AKI). Patients with AoCKI often face significantly heightened mortality, increased dependence on renal replacement therapy (RRT), and reduced likelihood of renal function recovery. Crucially, the pathophysiological mechanism of AoCKI is not merely an additive effect of two separate conditions, but a distinct synergistic insult driven by pre-existing microvascular and metabolic vulnerabilities. While recent clinical epidemiological studies have enhanced understanding of AoCKI, the mechanisms underlying its poor prognosis remain insufficiently elucidated, and research on population-specific biomarkers is limited. Most currently available biomarkers were identified in patients with isolated AKI and have not been specifically studied in the context of CKD. This review synthesizes previous findings and relevant research to examine the risk factors for AoCKI, as well as the potential pathophysiological mechanisms and biomarkers associated with impaired renal adaptive repair. The objective is to inform the development of more effective preventive measures, diagnostic criteria, and treatment strategies to improve outcomes for this patient population.

## 1 Introduction

Chronic kidney disease (CKD) and acute kidney injury (AKI) were previously regarded as two distinct clinical syndromes; however, recent clinical studies increasingly demonstrate a close relationship between them (Chawla and Kimmel, 2012; Chawla et al., 2014). Following the onset of AKI, kidneys frequently develop irreversible chronic damage due to inadequate repair mechanisms (Coca et al., 2012; Sawhney et al., 2015). Conversely, pre-existing CKD is recognized as a major risk factor for AKI (James et al., 2015). CKD patients are susceptible to various precipitating factors, which can lead to a short-term decline in glomerular filtration rate and subsequent development of AKI. The inherent fragility of the kidneys in CKD patients increases their sensitivity to external adverse stimuli. Epidemiological data confirmed this heightened susceptibility (Johansen et al., 2024), and recent registries indicate that a vast majority of patients with AKI have an underlying history of CKD (Nash et al., 2002). In a clinical study of 1,005 patients, Zhou et al. found that the incidence of AoCKI was higher than that of new-onset AKI (Zhou et al., 2012). Furthermore, the risk of developing AKI escalates exponentially with advancing CKD stages, with patients exhibiting lower baseline estimated glomerular filtration rates (eGFR) facing a substantially higher likelihood of acute functional decline (Alabdan et al., 2017; Hsu et al., 2008).

**FIGURE 1:**
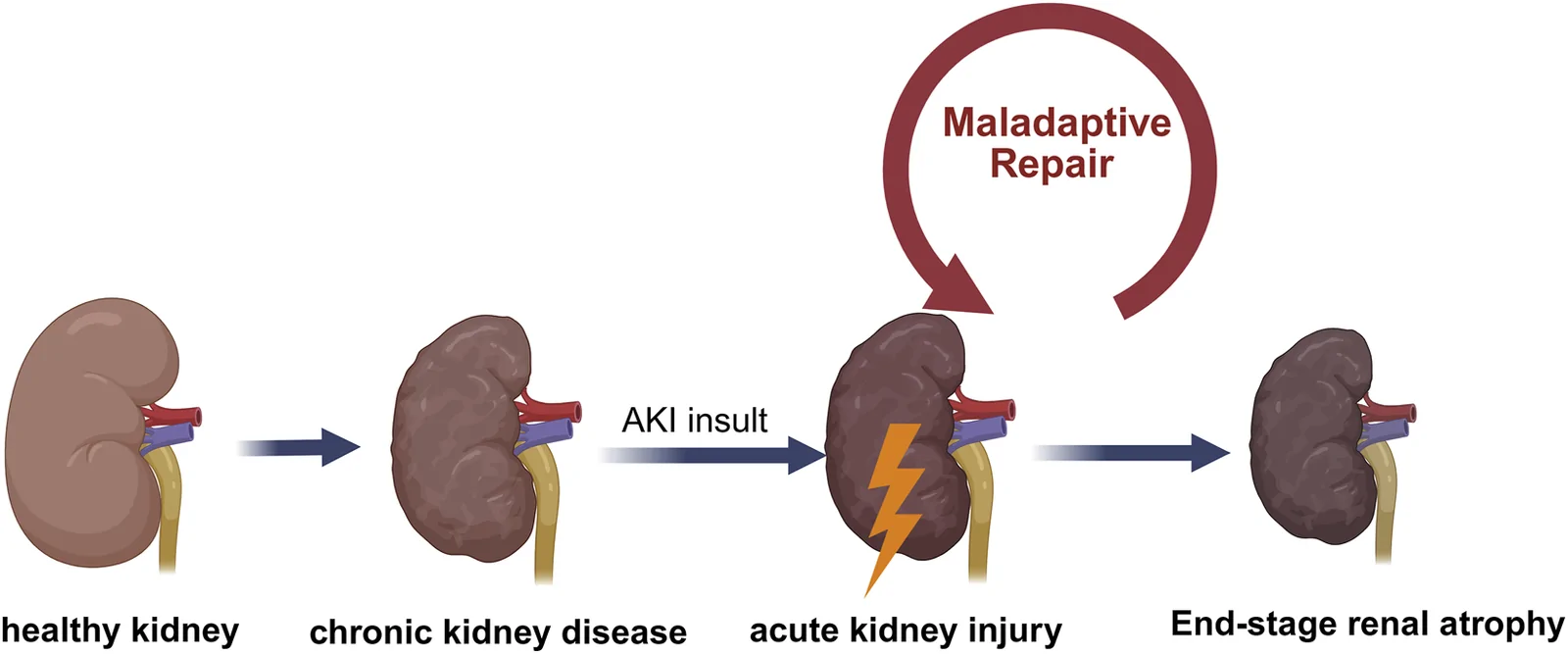
Kidneys with pre-existing chronic damage are more susceptible to entering a vicious cycle of maladaptive repair following a secondary AKI insult, ultimately accelerating the progression toward irreversible end-stage atrophy and renal failure.

Compared with isolated AKI, AoCKI is often associated with a poorer prognosis. Animal studies have demonstrated that CKD rats experience a markedly shorter mean survival time following ischemia-reperfusion injury than rats with normal renal function (71.0 ± 7.1 min versus 112.4 ± 11.0 min, p < 0.05) (Skott et al., 2010). Several large-scale clinical cohort studies corroborate these findings. For instance, a large-scale longitudinal studies reveal that the risk of progression to end-stage renal disease (ESRD) and overall mortality surges in patients with lower pre-morbid eGFR prior to the AKI onset (Pannu et al., 2011). This accelerated trajectory toward renal failure is starkly evident, with recent cohorts reporting that mortality rates can approach 30% and a significant proportion of patients require immediate renal replacement therapy (RRT) (Chagas et al., 2026). The risk of death can increase nearly 17-fold within months following the acute episode, and complete renal recovery is often delayed or unattained (Hamroun et al., 2022). The adaptive repair response of the CKD kidney is very fragile, often characterized by altered renal hemodynamics and increased intraglomerular pressure resulting from compensatory hyperfiltration by residual nephrons. This vulnerability is exacerbated during and after AKI episodes, creating a cycle of injury and maladaptation (Basile et al., 2016; Zoccali et al., 2025) (Figure 1) (Table 1).

**TABLE 1:** Comparison of the clinical characteristics of CKD, AKI and AoCKI.

Characteristics	CKD	AKI	AoCKI
Definition	Progressive decline in renal function over >3 months	Abrupt decline in renal function	AKI occurring on pre-existing CKD
Major pathological feature	Chronic fibrosis, nephron loss	Acute tubular injury	Acute injury superimposed on maladaptive repair background
Main risk factors	Diabetes, hypertension, obesity	Sepsis, surgery, nephrotoxic drugs	CKD + infection + surgery + cardiovascular disease + nephrotoxins
Representative biomarkers	eGFR, albuminuria	NGAL, KIM-1, TIMP-2×IGFBP7	NGAL, KIM-1, TIMP-2×IGFBP7, PENK, FGF23
Recovery potential	Slow progression	Usually recoverable	Limited adaptive repair
Recovery rate	Variable	High (depending on severity)	Lower than isolated AKI
Mortality	Moderate	Increased	Highest
Long-term outcome	ESRD	AKI-CKD transition	Rapid progression to ESRD
Current therapy	CKD management	Supportive treatment	Mainly supportive treatment; no disease-specific therapy

Beyond progressive impairment of renal function, CKD is increasingly recognized as a multisystem disorder characterized by chronic low-grade inflammation, immune dysregulation, metabolic disturbances, vascular dysfunction, and accelerated biological ageing. Consequently, patients with CKD frequently present with multimorbidity, including cardiovascular disease, diabetes mellitus, frailty, osteoporosis, and osteoarthritis. These comorbidities not only share common inflammatory and metabolic pathways with CKD but also further compromise renal resilience to acute insults. For example, osteoarthritis is highly prevalent in patients with CKD, and chronic systemic inflammation, reduced physical activity, as well as the frequent use of nonsteroidal anti-inflammatory drugs (NSAIDs) may collectively increase the susceptibility to AKI. Therefore, AoCKI should be considered within the broader context of CKD multimorbidity rather than as an isolated renal event, as this perspective may better explain the heterogeneity of disease progression and clinical outcomes. Therefore, understanding AoCKI within the broader context of CKD multimorbidity may provide new insights into the mechanisms of maladaptive repair, facilitate the identification of clinically relevant biomarkers, and promote the development of targeted therapeutic strategies.

## 2 Risk factors of AKI superimposed on CKD

### 2.1 Serious infection

Infection represents a major contributor to the progression of CKD and serves as a primary trigger for AKI. CKD patients are at a substantially increased risk of infection due to immune dysfunction, malnutrition, and greater exposure to infectious agents during hospitalization (Syed-Ahmed and Narayanan, 2019). Epidemiological evidence indicates that 25%–75% of AKI cases globally are associated with sepsis or septic shock (Poston and Koyner, 2019; Zhi et al., 2019; Zarbock et al., 2023a). A retrospective cohort study involving approximately 85,000 critically ill patients demonstrated that pre-existing CKD independently doubles the risk of developing infection-associated AKI compared to patients with normal baseline renal function (White et al., 2024). Furthermore, sepsis-associated AKI not only occurs frequently but is also linked to poor prognosis. A new large cohort study reported that among patients hospitalized for sepsis, 42.2% developed AKI, and 17.6% of these cases progressed to persistent acute kidney disease (AKD) (Chiang et al., 2025). These findings indicate that infection-induced kidney injury can evolve from an acute phase to a chronic, irreversible pathological process. Sepsis-associated AKI is further correlated with adverse clinical outcomes, including prolonged hospital stays, higher mortality rates, increased long-term disability, and diminished quality of life (Shum et al., 2016; Peerapornratana et al., 2019). Consequently, prevention and early identification of infections are essential for mitigating adverse renal outcomes.

### 2.2 Nephrotoxic drugs

Drug-induced AKI(DI-AKI) constitutes a substantial proportion of both community-acquired AKI (CA-AKI) and hospitalized patients (Perazella, 2009; 2018). For CKD patients who already have reduced renal reserve, the risk associated with nephrotoxic drugs is significantly heightened. Evidence indicates that, even with standard preventive measures, the risk of contrast-induced nephropathy (CIN) in CKD patients is approximately 85% higher than in those with normal renal function (OR = 1.858, 95% CI: 1.105–3.125, p = 0.0023) (Olariu et al., 2025). Nephrotoxic drugs primarily induce kidney injury through three mechanisms: direct tubular damage, intratubular obstruction, and local inflammation (Perazella and Rosner, 2022). Stucke et al. reported that, among 352 patients with a history of CKD, diuretic use was an independent risk factor for CA-AKI (OR = 1.82, 95% CI: 1.0–3.04) (Stucker et al., 2017). Renin-angiotensin system inhibitors (RASi), commonly prescribed for CKD patients, may precipitate AKI during periods of hemodynamic instability (Alabdan et al., 2017; Assaly et al., 2023). Additionally, several commonly used nephrotoxic medications require particular caution in CKD patients. These include antibiotics such as vancomycin (Kan et al., 2022) and aminoglycosides (Lopez-Novoa et al., 2011), iodinated contrast agents used in imaging procedures (Aggarwal et al., 2025; Herath et al., 2025), non-steroidal anti-inflammatory drugs (NSAIDs) (Whelton, 1999), and calcineurin inhibitors. Vancomycin and aminoglycosides induce tubular epithelial injury through direct cytotoxic effects and oxidative stress (Lopez-Novoa et al., 2011; Elyasi et al., 2012), whereas iodinated contrast agents contribute to renal vasoconstriction, medullary hypoxia, and tubular injury (Azzalini et al., 2016). NSAIDs impair renal autoregulation through prostaglandin inhibition, and calcineurin inhibitors promote vasoconstriction and chronic tubulointerstitial injury. While exposure to nephrotoxic drugs is necessary for the development of drug-induced AKI (DI-AKI), drug properties alone do not account for nephrotoxicity; the patient’s underlying renal condition is also a significant contributing factor (Perazella, 2018; Garcia et al., 2024).

### 2.3 Cardiovascular diseases

There is a close functional relationship between the heart and the kidneys (Lekawanvijit and Krum, 2014; Schefold et al., 2016; Lee et al., 2018). Cardiovascular disease is a common complication among CKD patients. Several studies have demonstrated that a decline in glomerular filtration rate (GFR) in CKD patients can accelerate the progression of cardiovascular disease and heart failure; notably, the risk of developing cardiovascular disease in CKD patients exceeds the risk of progression to end-stage renal disease (Jankowski et al., 2021). Conversely, cardiovascular events are a major trigger for AKI. Studies indicate that approximately 20%–40% of patients with acute decompensated heart failure develop AKI (Heywood et al., 2007). Furthermore, one study reported that CKD patients have a higher risk of developing AKI following ST-segment elevation myocardial infarction (STEMI) compared to non-CKD patients; specifically, patients with stage 3 b CKD or later have a threefold higher risk of AKI than other patients (Schefold et al., 2016).

### 2.4 Surgery

Surgery is one of the major risk factors for AKI in patients with CKD. A retrospective study of CKD patients undergoing cardiac surgery showed that 50% of patients developed cardiac surgery-associated acute kidney injury (CSA-AKI) (Zhang et al., 2023). Intraoperative hypotension and inadequate renal perfusion are direct triggers of AKI, and patients with CKD have poorer tolerance to blood pressure fluctuations due to impaired renal autoregulation, making them more susceptible to pre-renal AKI. A prospective, international, multicenter observational study enrolled 10,568 patients undergoing major surgery from 30 countries, the results showed that the overall incidence of postoperative AKI (PO-AKI) was 18.4%, and CKD was an independent risk factor for PO-AKI, pre-existing CKD significantly increased the risk of PO-AKI (OR 2.0, 95% CI 1.63–2.45, p < 0.001) (Zarbock et al., 2023b). In addition, complications such as systemic inflammatory response syndrome (SIRS) induced by cardiopulmonary bypass, the use of nephrotoxic medications during the perioperative period (e.g., antibiotics, nonsteroidal anti-inflammatory drugs, and contrast agents), and postoperative infections may all cause cumulative damage to the residual renal function of patients with CKD.

### 2.5 Chronic diseases

Obesity, diabetes, and hypertension are also risk factors for AoCKI. Obesity exacerbates the course of many primary kidney diseases, such as glomerulonephritis and chronic kidney injury. Microalbuminuria, proteinuria, high glomerular filtration rate, impaired renal function, and structural changes in the kidneys are also associated with elevated BMI. This may be due to an increased risk of impaired gastrointestinal barrier function in obese patients, leading to increased local serosal fluid accumulation and the malignant spread of toxins and bacteria. The kidneys’educed ability to clear inflammatory mediators consequently results in renal injury (Amann and Benz, 2013). CKD patients often have comorbidities such as diabetes, hyperlipidemia, hypertension, and hyperuricemia, all of which are independent risk factors for AKI. Hsu et al. found that, compared to patients who did not develop AKI, those with AKI had a higher prevalence of pre-existing chronic conditions such as diabetes and hypertension (Hsu et al., 2008). This suggests that, in addition to monitoring kidney function and treating kidney disease, we must also closely monitor the underlying conditions of patients with CKD.

## 3 Core mechanism underlying the poor prognosis of AoCKI: maladaptive repair

After the onset of AKI, damaged renal tubular epithelial cells (TECs) frequently enter a maladaptive repair state (Ferenbach and Bonventre, 2015; Venkatachalam et al., 2015). While some TECs survive and proliferate, they often fail to redifferentiate adequately to restore normal structure and function. Instead, these cells remain dedifferentiated and acquire a pro-inflammatory, pro-fibrotic secretory profile known as the senescence-associated secretory phenotype (SASP), which serves as a central pathological driver of CKD progression. Cells exhibiting SASP continuously release pro-inflammatory and pro-fibrotic mediators. Concurrently, the physiological secretion of essential angiogenic factors, such as vascular endothelial growth factor (VEGF), is drastically downregulated. This critical paracrine imbalance drives the proliferation of interstitial pericytes and their transdifferentiation into matrix-producing myofibroblasts (Polichnowski, 2018). Additionally, these signals disrupt pericyte-endothelial cell interactions, causing pericyte detachment from the capillary wall and subsequent endothelial cell injury and microvascular rarefaction.

### 3.1 The vulnerable baseline: microvascular rarefaction and tissue hypoxia

**FIGURE 2:**
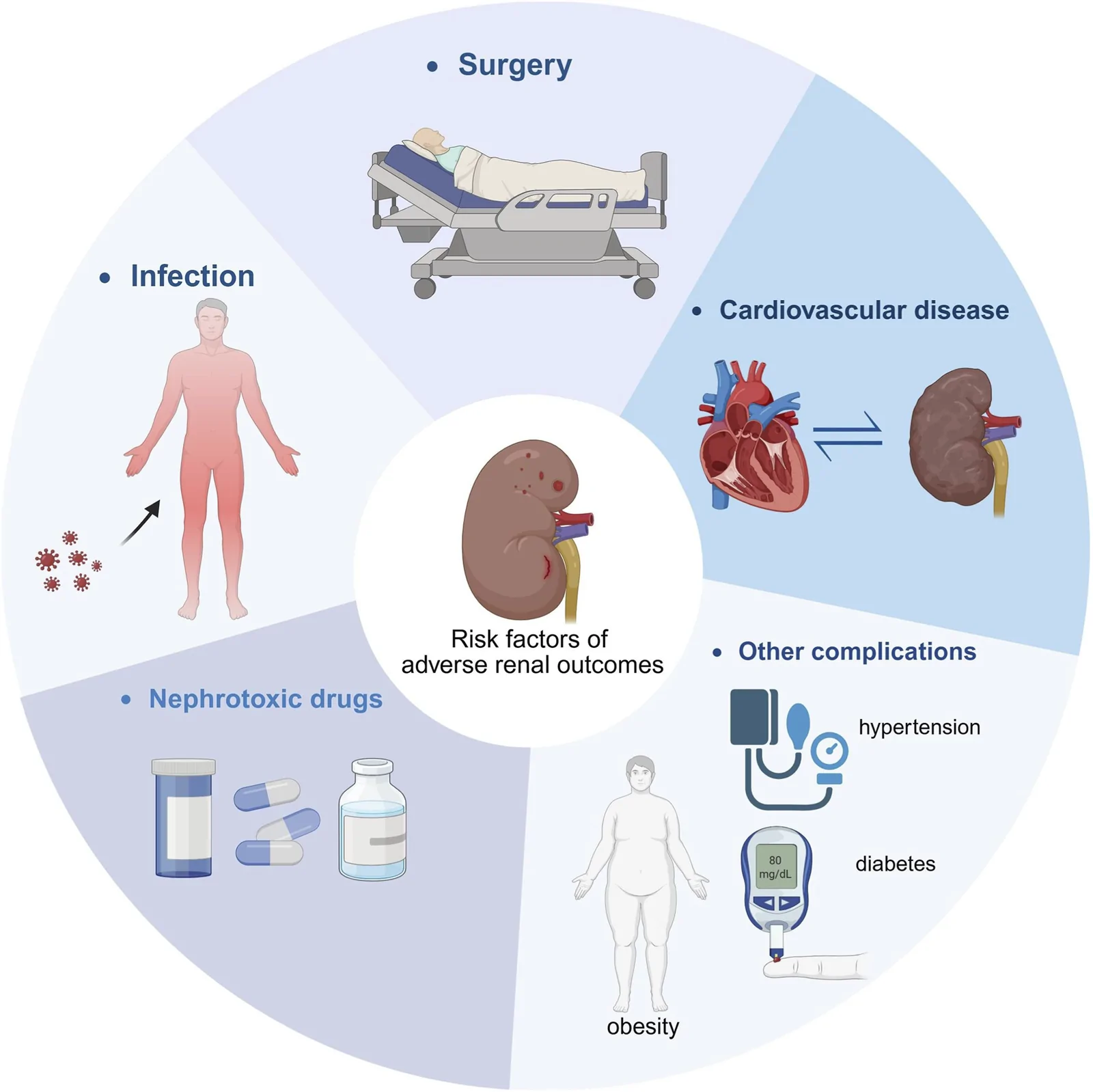
Risk factors of Acute-on-chronic kidney injury.

Microvascular rarefaction is a critical determinant of poor prognosis in AoCKI. CKD patients already demonstrate renal microvascular sparsity, which impairs the restoration of normal structure and function in damaged TECs(Polichnowski, 2018). Pre-existing microvascular sparsity interacts with new vascular injury following AKI, resulting in a synergistic effect (Mattson, 2003; Pechman et al., 2009). In a CKD rat model with 75% nephrectomy, one study found that although the initial severity of AKI was comparable to controls, CKD rats developed more pronounced microvascular sparsity after 4 weeks. These sparse regions were adjacent to dedifferentiated tubules that continued to express beta-catenin (Polichnowski et al., 2014). These findings indicate that the kidney’s vascular repair capacity is already compromised in CKD, hindering the restoration of a normal capillary network after exposure to additional risk factors. A sparse capillary network leads to severe and persistent local tissue hypoxia, which induces growth arrest, oxidative stress, and metabolic abnormalities in renal tubular epithelial cells (Basile et al., 2012; Ferenbach and Bonventre, 2015; Ullah and Basile, 2019), thereby disrupting normal repair processes. Additionally, nephron loss and impaired renal blood flow autoregulation due to microvascular sparsity act synergistically with the hypertension commonly observed in CKD. Even mild elevations in blood pressure can further aggravate glomerulosclerosis and interstitial fibrosis through pressure-transmission-mediated mechanical injury (barotrauma) (Polichnowski, 2018). Microvascular sparsity and tissue hypoxia, as both histological outcomes and sustaining factors of maladaptive repair, may be further intensified by upstream cellular and molecular mechanisms discussed below. Conversely, tissue hypoxia can negatively impact the cell cycle, mitochondrial function, and chronic inflammation, establishing a vicious cycle that persistently impedes renal repair (Figure 2).

### 3.2 Cellular and molecular mechanisms of maladaptive repair

#### 3.2.1 Cell cycle arrest

Cell cycle arrest represents a central aspect of maladaptive repair in TECs. In AKI, G2/M phase arrest is recognized as a primary driver of renal fibrosis (Yang et al., 2010). Notably, many chemotherapeutic agents are direct inducers of G2/M arrest, making this pathway clinically relevant to anticancer drug-induced AKI. Cisplatin, one of the most widely used platinum-based chemotherapeutics, causes DNA cross-linking and replication stress in proximal tubular epithelial cells, activating the DNA damage response and driving cells into G2/M arrest alongside oxidative stress and mitochondrial dysfunction (Liu et al., 2025). At the molecular level, cisplatin-induced upregulation of the m6A methyltransferase ZC3H13 stabilizes NABP1 mRNA and promotes G2/M arrest and apoptosis in tubular cells, while inhibition of ZC3H13 alleviates cell cycle arrest and renal injury in cisplatin-treated mice, illustrating G2/M arrest as a targetable mechanism in chemotherapy-induced AKI(Sheng et al., 2025). Beyond the acute setting, repeated low-dose cisplatin and other G2/M-arresting agents such as paclitaxel can drive kidney fibroblasts toward a senescent, myofibroblast-like phenotype, linking chemotherapy-induced G2/M arrest to long-term CKD development (Yu et al., 2022). Other conventional anticancer agents, including ifosfamide and pemetrexed, are likewise recognized causes of AKI, underscoring that G2/M-mediated tubular injury from cytotoxic chemotherapy is a broadly relevant driver of AKI and its progression to CKD in cancer populations (Pabla and Dong, 2008).

However, in AKI models with a CKD background, studies indicate that TECs predominantly undergo a sustained G1 phase arrest (Lim et al., 2018). Chronic metabolic stress in pre-existing CKD maintains the p53/p21 pathway in a pre-activated state. After the acute insult of AKI, expression levels of this pathway remain elevated during the recovery phase, thereby preventing successful progression through the G1/S checkpoint and resulting in prolonged G1 arrest (Goyal et al., 2024). Renal tubular epithelial cells arrested in the G1 phase develop a pro-fibrotic phenotype, marked by increased TGF-β expression, which promotes fibrotic processes and extracellular matrix deposition. These findings suggest that p53-mediated G1 phase arrest could serve as a potential therapeutic target for poor prognosis in AoCKI. The preactivation of the p53/p21 pathway in CKD may increase the sensitivity of tubular cells to acute injury and predispose them to persistent G1 phase arrest, thereby favoring fibrosis over regenerative repair.

#### 3.2.2 Chronic inflammation and dysregulation of the immune microenvironment

CKD patients exist in a state of chronic low-grade inflammation, characterized by continuous immune cell infiltration and elevated basal levels of TNF-α, IL-6, and IL-1β. (He et al., 2017; Fu et al., 2022; Kadatane et al., 2023). This persistent inflammatory state increases susceptibility to AKI and impairs renal recovery. Elevated pro-inflammatory factors in CKD are associated with a higher risk of cardiovascular events (Annuk et al., 2005; Borges et al., 2016), reduced renal blood flow, and decreased tolerance to ischemic conditions, thereby further increasing the risk of AKI(He et al., 2017). Following AKI onset, damaged renal tubular epithelial cells release large quantities of damage-associated molecular patterns (DAMPs), which amplify the inflammatory cascade through activation of Toll-like receptors (TLRs) and the NLRP3 inflammasome (Zoccali et al., 2025; Geng et al., 2026). This cascade results in excessive recruitment and activation of M1 macrophages, leading to further release of pro-inflammatory mediators. The sustained inflammatory microenvironment not only induces direct damage and apoptosis in renal tubular epithelial cells but also promotes renal interstitial fibrosis by activating fibroblasts and enhancing extracellular matrix deposition (Zhang et al., 2024). In individuals with pre-existing CKD, the chronic inflammatory state leads to persistent immune cell recruitment and activation, which further impairs renal recovery and accelerates progression to end-stage renal disease.

Emerging evidence suggests that thrombospondin-1 (TSP1) represents a critical upstream regulator linking hypoxia, endothelial dysfunction, and fibrosis during AoCKI(Sweetwyne and Murphy-Ullrich, 2012). TSP1 is a matricellular glycoprotein that is markedly upregulated in response to hypoxia, oxidative stress, and tissue injury. Importantly, TSP1 is recognized as the principal physiological activator of latent transforming growth factor-β (TGF-β), promoting the release of bioactive TGF-β from its latent complex and thereby initiating downstream Smad-dependent profibrotic signalling (Yang et al., 2018). Beyond TGF-β activation, TSP1 contributes directly to endothelial dysfunction, capillary rarefaction, inflammatory cell recruitment, and extracellular matrix deposition, all of which are hallmarks of maladaptive tubular repair. Recent studies therefore suggest that the TSP1–TGF-β signalling axis functions as a central molecular bridge linking persistent hypoxia and chronic inflammation to progressive fibrosis and CKD progression (Miguel et al., 2025). Given its upstream regulatory role, targeting the TSP1–TGF-β pathway may represent a promising therapeutic strategy to interrupt maladaptive repair and delay the transition from AKI to CKD in patients with AoCKI.

#### 3.2.3 Mitochondrial dysfunction

The kidneys possess the second-highest mitochondrial content and oxygen consumption in the human body, following the heart (Ralto et al., 2020). As a result, the pathogenesis of numerous kidney diseases is closely linked to renal mitochondrial dysfunction (Carney, 2014; Liu et al., 2019). In CKD, renal TECs demonstrate reduced mitochondrial biogenesis, as indicated by downregulated PGC-1α levels, imbalanced mitochondrial homeostasis characterized by increased fission and impaired fusion, and compromised oxidative phosphorylation function (Sharma et al., 2013; Zhan et al., 2015). These structural and functional mitochondrial impairments increase the vulnerability of renal tissue to AKI and impede renal recovery from AKI(He et al., 2017). Mitochondrial energy depletion and NAD^+^ depletion can result in SIRT1/3 inactivation, which subsequently induces cell cycle arrest in renal tubular epithelial cells through the p53/p21 pathway, thereby inhibiting regenerative repair68. Impaired mitochondria also generate excessive reactive oxygen species (ROS), surpassing the clearance capacity of the already compromised antioxidant system and causing oxidative damage to lipids, proteins, and DNA. Additionally, mitochondrial damage triggers robust inflammatory responses by releasing damage-associated molecular patterns (DAMPs), such as mitochondrial DNA (mtDNA), which activate the cGAS-STING pathway and continuously stimulate the NLRP3 inflammasome and innate immune responses (Gritsenko et al., 2020). Ultimately, these processes lead to fibroblast activation and extracellular matrix deposition, promoting renal interstitial fibrosis.

**FIGURE 3:**
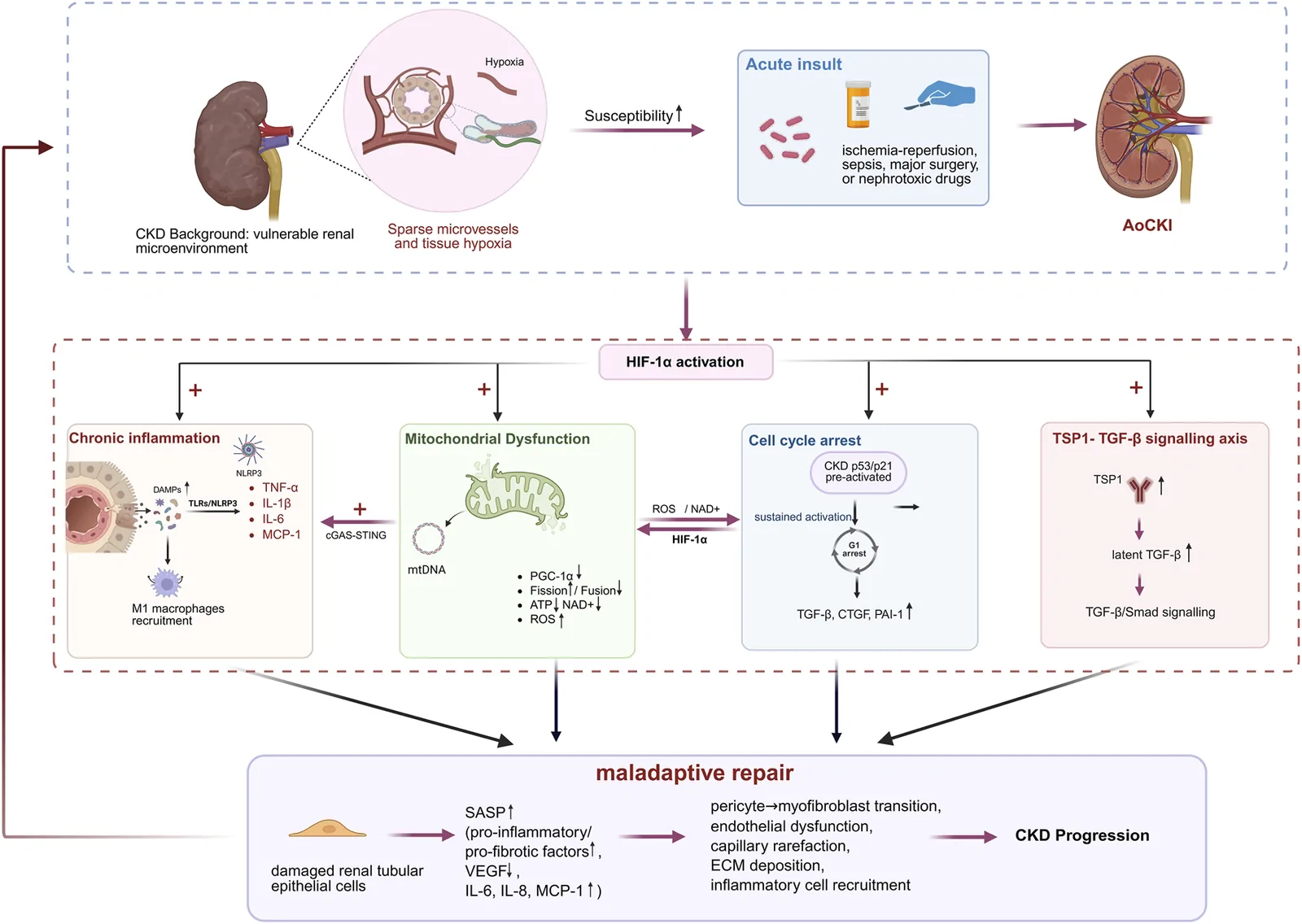
Integrated mechanistic framework underlying maladaptive repair and disease progression in AoCKI. Pre-existing CKD establishes a vulnerable renal microenvironment characterized by capillary rarefaction and chronic hypoxia. Following a secondary acute insult (e.g., ischemia, sepsis, surgery, or nephrotoxic drugs), persistent HIF-1α activation triggers interconnected pathological pathways, including mitochondrial dysfunction, oxidative stress, chronic inflammation, sustained cell-cycle arrest, and TSP1-mediated activation of TGF-β signaling. These mutually reinforcing mechanisms promote maladaptive tubular repair, progressive fibrosis, endothelial dysfunction, and further microvascular rarefaction, resulting in CKD progression and increased susceptibility to recurrent AKI, thereby establishing a self-perpetuating vicious cycle. Abbreviations: AoCKI, Acute-on-chronic kidney injury; ATP, Adenosine triphosphate; cGAS-STING, Cyclic GMP-AMP synthase–stimulator of interferon genes; CKD, Chronic kidney disease; CTGF, Connective tissue growth factor; DAMPs, Damage-associated molecular patterns; ECM, Extracellular matrix; HIF-1α, Hypoxia-inducible factor 1-alpha; IL-1β, Interleukin-1 beta; IL-6, Interleukin-6; IL-8, Interleukin-8; MCP-1, Monocyte chemoattractant protein-1; mtDNA, Mitochondrial DNA; NAD^+^, Nicotinamide adenine dinucleotide; NLRP3, NOD-, LRR- and pyrin domain-containing protein 3; PAI-1, Plasminogen activator inhibitor-1; PGC-1α, Peroxisome proliferator-activated receptor gamma coactivator 1-alpha; ROS, Reactive oxygen species; SASP, Senescence-associated secretory phenotype; TGF-β, Transforming growth factor-beta; TLRs, Toll-like receptors; TNF-α, Tumor necrosis factor-alpha; TSP1, Thrombospondin-1; VEGF, Vascular endothelial growth factor).

Although these mechanisms are described separately in the following sections, they do not operate independently during AoCKI. Rather, they form a highly interconnected pathological network centered on maladaptive repair. Pre-existing CKD establishes a metabolically and structurally vulnerable renal microenvironment characterized by nephron loss, microvascular rarefaction, and chronic hypoxia, which predisposes the kidney to maladaptive responses following a secondary AKI insult (Baker and Cantley, 2025). Mitochondrial dysfunction not only impairs ATP production but also promotes excessive ROS generation and mtDNA release, thereby amplifying inflammatory signaling through innate immune pathways (Cao et al., 2025). In turn, chronic inflammation further aggravates mitochondrial dysfunction by disrupting mitochondrial biogenesis and oxidative phosphorylation. Oxidative stress simultaneously activates the p53/p21 pathway, resulting in sustained tubular epithelial cell-cycle arrest and acquisition of the senescence-associated secretory phenotype, which further amplifies inflammatory and profibrotic signaling. These reciprocal interactions perpetuate endothelial dysfunction and capillary rarefaction, thereby worsening tissue hypoxia and reinforcing HIF-1α activation (Miguel et al., 2025). Consequently, the kidney becomes trapped in a self-amplifying cycle of maladaptive repair, ultimately leading to progressive fibrosis, CKD progression, and increased vulnerability to recurrent AKI (Figure 3).

## 4 Potential biomarkers associated with the prognosis of AoCKI

### 4.1 Neutrophil gelatinase-associated Lipocalin (NGAL)

Neutrophil gelatinase-associated lipocalin (NGAL) is a 25-kDa protein belonging to the lipocalin superfamily that is expressed in various tissues throughout the body, such as the lungs, gastrointestinal tract, liver, and kidneys (Paragas et al., 2012). NGAL is an important biomarker for the early diagnosis of AKI, in recent years, there has been growing interest in its predictive value for AoCKI. Doi et al. found that preoperative plasma NGAL levels in patients with CKD were significantly associated with the risk of postoperative AoCKI following cardiac surgery (Doi et al., 2013). Notably, this study observed a transient decrease in plasma NGAL levels 0–4 h postoperatively in patients with AoCKI, suggesting the possible existence of an as-yet-unelucidated mechanism of endogenous NGAL release inhibition or increased uptake under the stress of cardiac surgery. The time to NGAL peak occurs earlier than that of serum creatinine, indicating a temporal advantage in predicting AKI recovery. In the context of CKD, the interference of baseline renal function on NGAL diagnostic thresholds cannot be overlooked. An analysis by Banai et al. of 217 patients with ST-segment elevation myocardial infarction (STEMI) undergoing emergency percutaneous coronary intervention (PCI) found that patients with CKD required higher NGAL cutoff values to achieve optimal diagnostic performance: The optimal cutoff value for predicting AKI was 104 ng/ml (AUC 0.844) in patients without CKD, whereas it was 133 ng/ml in patients with CKD (Banai et al., 2021). This finding underscores the need to account for the influence of baseline renal function when interpreting NGAL levels in the context of CKD. Kim et al. introduced the concept of ΔNGAL (change over 48 h) and found that ΔNGAL was significantly higher in the AKI group than in the AoCKI group (159.5 ± 261.5 ng/ml vs. 10.1 ± 229.7 ng/ml, P = 0.001), suggesting that dynamic changes in NGAL levels may aid in distinguishing between simple AKI and AoCKI as two distinct injury types (Kim et al., 2019); Jiang et al.‘s animal study found that, compared to the kidneys of mice with simple CKD, both NGAL mRNA and protein levels were significantly elevated in the kidneys of mice with severe AoCKI (Jiang et al., 2021).

### 4.2 Kidney injury molecule 1 (KIM-1)

Kidney Injury Molecule-1 (KIM-1) is a type I transmembrane glycoprotein that is expressed at very low levels in normal renal tissue; however, it is highly expressed in renal tubular epithelial cells when the kidney is damaged, such as in cases of ischemia-reperfusion injury, drug-induced toxicity, or renal transplant rejection (Han et al., 2002; Yang et al., 2015). KIM-1 not only serves as a biomarker capable of sensitively predicting the onset of AKI but is also closely associated with the progression of AKI and CKD (Matarneh et al., 2025). Notably, the magnitude and kinetics of KIM-1 elevation differ between AKI and CKD. In a cross-sectional comparison of patients with various acute and chronic kidney diseases, Han et al. found that urinary KIM-1 concentrations were significantly higher in patients with AKI than in those with CKD or in healthy controls, and that KIM-1 rose earlier than serum creatinine in a prospective cohort undergoing cardiopulmonary bypass surgery (Han et al., 2008). In contrast, KIM-1 elevation in stable CKD tends to be more modest and tracks the degree of eGFR impairment rather than an acute injury event: in the large NURTuRE-CKD cohort, median plasma KIM-1 was 250 pg/mL and median urinary KIM-1 was 178 pg/mmol creatinine, with both biomarkers rising progressively across worsening eGFR categories and correlating inversely with baseline eGFR (McDonnell et al., 2025). Consistent with this, KIM-1 has been shown to remain elevated for a prolonged period even after apparent functional recovery from an AKI episode, indicating persistent subclinical tubular injury that may bridge the acute and chronic states (Cuesta et al., 2022). Together, these findings suggest that a sharp, transient rise in KIM-1 favors a diagnosis of AKI, while a sustained, lower-grade elevation is more indicative of chronic, ongoing tubular injury in CKD, and that in the acute-on-chronic setting, both the absolute level and the trajectory of KIM-1 over time may carry distinct prognostic information.

KIM-1 levels play a bidirectional role in the progression of kidney injury. In animal studies, Jiang et al. found that KIM-1 mRNA levels rose sharply in mild AoCKI; whereas in severe acute-on-chronic kidney injury, KIM-1 expression showed no significant difference (Jiang et al., 2021), his may be because, in mild AoCKI, KIM-1 initiates potential renal repair; KIM-1 exerts a protective effect on the kidneys by promoting the repair of renal tubular epithelial cells through enhanced cell migration and proliferation (Zhang and Cai, 2016); If the injury is too severe to trigger repair, KIM-1 levels will not rise. This finding has important implications for the clinical interpretation of AoCKI: in CKD patients following AKI, a moderate elevation in KIM-1 may indicate that the kidney still possesses reparative capacity; conversely, the absence of KIM-1 elevation suggests that the kidney has lost its reparative potential, indicating a poorer prognosis. Persistent, chronic elevation of KIM-1 expression may lead to adverse renal pathological processes. If tubular injury persists without completion of repair, long-term KIM-1 expression can promote chronic inflammation and interstitial fibrosis through various mechanisms, leading to the progression of CKD (Humphreys et al., 2013; Zhang and Liu, 2025). A recent study by Xiao et al. revealed the potential of KIM-1 as a therapeutic target. By constructing KIM-1-targeted degrading liposomes (KIM1-TDL) and utilizing a modified PROTAC strategy, they achieved targeted degradation of the KIM-1 protein in cisplatin-induced AKI cell and mouse models. The results showed that KIM1-TDL significantly reduced injury-induced KIM-1 protein levels (with degradation efficiencies ranging from 71.8% to 81.3%), improved renal function, reduced renal tubular epithelial cell apoptosis, and suppressed the expression of inflammatory factors, increasing the 5-day survival rate of AKI mice from 10% to 90% (Xiao et al., 2026). This suggests that targeted degradation of KIM-1 can effectively alleviate AKI and improve prognosis.

### 4.3 Tissue inhibitor metalloproteinases-2 (TIMP-2) and insulin-like growth factor-binding protein 7

Tissue Inhibitor of Metalloproteinases-2 (TIMP-2) and Insulin-like Growth Factor-binding Protein 7 (IGFBP7) are both key mediators of cell cycle arrest. When renal tubular epithelial cells are exposed to stressors such as ischemia, hypoxia, or inflammation, they activate the p53/p21 and TGF-β pathways, thereby upregulating the expression of TIMP-2 and IGFBP7. This blocks the function of the cyclin-dependent kinase complex, inducing cells to temporarily arrest in the G1 phase to prevent the replication of damaged DNA and allow time for repair (Kashani et al., 2013; Liu et al., 2016). Clinically, urinary [TIMP-2]·[IGFBP7] has been demonstrated to be a reliable predictor of moderate-to-severe AKI in critically ill patients and has been approved by the FDA (the U.S. Food and Drug Administration) for the clinical prediction of AKI(Ilaria et al., 2021). In the complex context of AoCKI, the expression profiles of these two markers exhibit nonlinear characteristics, and their levels correlate with the severity of AKI. In an animal study of AoCKI, compared with mice with CKD alone, IGFBP7 protein and TIMP-2 mRNA were moderately increased in kidneys with moderate AoCKI. However, in kidneys with severe AoCKI, accompanied by severe tubular necrosis and failed repair, IGFBP7 protein expression decreased significantly, while TIMP-2 protein expression further increased (Jiang et al., 2021). This suggests that the decrease in IGFBP7 protein levels may indicate the exhaustion of cellular defense mechanisms or massive shedding of renal tubular epithelial cells, foreshadowing the progression of AoCKI to irreversible damage. This indicates that the combination of TIMP-2 mRNA and IGFBP7 can serve as a biomarker for predicting AoCKI, and that the severity of kidney injury can be stratified based on their levels.

### 4.4 Proenkephalin (PENK)

Proenkephalin (PENK) is a stable polypeptide fragment generated by the cleavage of the precursor protein proenkephalin A (Denning et al., 2008). Because PENK has a low molecular weight and does not bind to plasma proteins, it is freely filtered through the glomeruli, its plasma concentration is inversely correlated with glomerular filtration rate (GFR) and can sensitively reflect dynamic changes in GFR (Marino et al., 2015). In the early detection of AKI, PENK often outperforms methods based on creatinine. Breidthardt et al. found in CKD patients undergoing contrast-enhanced imaging that there was no significant difference in baseline PENK levels between the AKI group and the non-AKI group. While a single measurement could not predict AKI, the predictive performance of ΔPENK was highly significant (AUC 0.92). Furthermore, compared to the traditional marker creatinine, ΔPENK could identify high-risk patients for AKI 24 h earlier (Breidthardt et al., 2018). In a study on AKI associated with cardiac surgery, Mossanen et al. found that elevated preoperative PENK levels were independently associated with the risk of postoperative AKI(Mossanen et al., 2017). Elevated PENK levels are associated with adverse renal clinical outcomes, including death and CKD progression, highlighting its predictive potential in renal prognosis. In a study with a median follow-up of 8.7 years, Würfel et al. found that for every twofold increase in baseline PENK, the risk of major adverse kidney events (including new-onset or worsening CKD, initiation of renal replacement therapy, and renal death) increased 2.14-fold (HR 2.14, 95% CI 1.26–3.61, p = 0.005) (Würfel et al., 2026). In patients with sepsis-associated AKI, Caironi et al. reported that PENK independently predicted future need for RRT (OR 4.0) and 90-day mortality (HR 1.5), and retained predictive utility even in patients with eGFR >70 (Caironi et al., 2018), indicating that PENK’s prognostic assessment is not dependent on baseline renal function. Currently, although numerous studies have demonstrated the association between PENK levels and the prognosis of AKI and CKD, there is inconsistency in the evaluation of its predictive utility in renal function. Further research is needed to validate its effectiveness across different stages of renal impairment and in various patient populations.

### 4.5 Fibroblast Growth Factor 23(FGF-23)

Fibroblast Growth Factor 23(FGF-23)is primarily secreted by osteoblasts and serves as a key regulator of calcium and phosphorus metabolism (Donate-Correa et al., 2014). The kidneys may clear some FGF23 through filtration and/or catabolism, which is why it has multiple pathophysiological implications in kidney disease (Pannu et al., 2011). FGF-23 has strong predictive power regarding the occurrence and prognosis of AKI. A prospective study by Cheruku et al. involving 173 cardiac surgery patients showed that for every twofold increase in cFGF-23 levels 6 h postoperatively, the risk of AKI increased by 57% (OR 1.57, p < 0.0001) (Cheruku et al., 2025). Recent studies have found a close association with the prognosis of AoCKI. A prospective study by Chang et al. involving 149 patients with acute-on-chronic kidney injury demonstrated that plasma cFGF-23 levels were an independent predictor of 90-day mortality (HR 2.5, 95% CI 1.5–4.1). This study was the first to confirm, in the specific A-on-C population, that cFGF-23 is a superior predictor of mortality compared to traditional tubular injury markers such as NGAL, KIM-1, TIMP-2, and IGFBP7(Chang et al., 2020). Fayed et al. found in 30 ICU-AKI patients that FGF23 levels were significantly higher in the mortality group than in the survival group (544.2 vs. 59.3 pg/mL, p = 0.004), with an AUC of 0.88 for predicting mortality; FGF-23 levels were also significantly elevated in the group requiring RRT (529.5 vs. 285.11 pg/mL, p = 0.04) (Fayed et al., 2019).

Calcium and phosphorus are not merely metabolites passively regulated by FGF-23, disturbances in calcium and phosphate signaling are themselves important contributors to the initiation and progression of AKI. At the cellular level, intracellular Ca^2+^ overload—driven by excessive Ca^2+^ influx through store-operated channels, aberrant Ca^2+^ release from the endoplasmic reticulum, and mitochondrial Ca^2+^ accumulation—triggers oxidative stress, mitochondrial permeability transition, and both necrotic and apoptotic tubular cell death, positioning Ca^2+^ signaling as a central determinant of renal cell fate during acute injury (Ning et al., 2021). Beyond intracellular signaling, extracellular calcium-phosphate supersaturation can itself be directly nephrotoxic: elevated calcium and phosphate concentrations promote the formation and intratubular deposition of calcium phosphate microcrystals, which injure tubular epithelial cells and activate the NLRP3 inflammasome, amplifying inflammatory and oxidative tubular damage and precipitating AKI (Yifan et al., 2020; Ukaeje et al., 2025). Systemically, acute hyperphosphatemia—arising from phosphate loading, tumor lysis, rhabdomyolysis, or reduced GFR-dependent phosphate clearance—further perturbs the interlinked PTH–FGF-23–vitamin D axis that governs calcium-phosphate homeostasis, creating a feed-forward loop in which impaired renal function raises serum phosphate, stimulates FGF-23 secretion, and in turn worsens tubular injury (Murray and Wolf, 2024). These mechanisms suggest that calcium and phosphate are active mediators, rather than passive markers, of AKI pathogenesis, offering biological plausibility for FGF-23 as a mechanistically grounded predictor of AKI occurrence and severity in the acute-on-chronic setting. Future studies should clarify the dynamic changes in FGF-23 levels in patients with acute-on-chronic kidney injury and investigate whether FGF-23-guided intervention strategies (such as phosphorus restriction and iron supplementation) can improve patient outcomes.

## 5 Emerging mechanism-based therapeutic strategies for AoCKI

Current management of AoCKI remains largely supportive, including optimization of hemodynamics, avoidance of nephrotoxic agents, and renal replacement therapy when indicated. However, these approaches do not directly target the molecular mechanisms responsible for maladaptive repair and progressive CKD. As increasing evidence indicates that persistent tubular injury, mitochondrial dysfunction, chronic inflammation, endothelial dysfunction, and fibrosis collectively drive the transition from AKI to CKD, mechanism-based therapeutic strategies have emerged as promising approaches to interrupt disease progression (Huang et al., 2023).

Targeting maladaptive tubular repair has become an important therapeutic strategy because persistent cell-cycle arrest and profibrotic signaling are central events promoting renal fibrosis. Modulating tubular epithelial cell repair, limiting senescence-associated secretory phenotypes, and restoring adaptive regeneration may reduce extracellular matrix accumulation and preserve renal function (Tang et al., 2021). Although these approaches remain largely experimental, they represent attractive targets for preventing irreversible structural remodeling after acute injury. Mitochondrial dysfunction is another promising therapeutic target. Mitochondria-directed antioxidants such as MitoQ and elamipretide (SS-31), together with agents that restore mitochondrial biogenesis through activation of the PGC-1α pathway or augmentation of NAD^+^ metabolism, have demonstrated renoprotective effects in preclinical models (McCully et al., 2026). These interventions reduce oxidative stress, improve ATP production, and attenuate tubular cell injury, thereby facilitating adaptive repair following AKI(Chang et al., 2024; Guo et al., 2024). Given the critical role of chronic inflammation in maladaptive repair, modulation of inflammatory pathways has also attracted increasing attention. Experimental studies have shown that inhibition of the NLRP3 inflammasome, blockade of pro-inflammatory cytokines, and regulation of macrophage polarization can attenuate renal inflammation and subsequent fibrosis. Rather than simply suppressing inflammation, these approaches aim to restore immune homeostasis and create a microenvironment favorable for tissue repair (Klinkhammer and Boor, 2023; Sanz et al., 2023).

Antifibrotic therapy represents another important direction for future AoCKI management. Because excessive activation of TGF-β signaling is a central driver of extracellular matrix deposition, several antifibrotic agents have been investigated to inhibit fibroblast activation and collagen synthesis (Murphy-Ullrich and Suto, 2018). Although clinical evidence remains limited, these therapies may help slow CKD progression by interrupting the maladaptive repair process. Among emerging therapeutic targets, the TSP1–TGF-β signaling axis has attracted particular interest because it functions as an upstream regulator linking chronic hypoxia, endothelial dysfunction, inflammation, and fibrosis. TSP1 activates latent TGF-β, thereby amplifying profibrotic signaling and promoting extracellular matrix accumulation (Sweetwyne and Murphy-Ullrich, 2012). Experimental studies suggest that inhibition of TSP1-mediated activation of latent TGF-β may preserve microvascular integrity, attenuate renal fibrosis, and improve adaptive tissue repair. Although these findings are currently confined primarily to experimental models, the TSP1–TGF-β pathway represents a promising mechanism-based therapeutic target for AoCKI and warrants further clinical investigation (Peek and Wilson, 2023).

Overall, future therapeutic strategies for AoCKI are unlikely to rely on a single intervention. Instead, combination therapies targeting mitochondrial dysfunction, chronic inflammation, maladaptive repair, and fibrosis may provide greater benefit than conventional supportive management alone. Continued integration of mechanistic insights with biomarker-guided patient stratification is expected to facilitate the development of precision therapies for AoCKI.

## 6 Conclusion

AKI superimposed on CKD constitutes a distinct clinical syndrome with unique pathophysiological features. Pre-existing renal alterations in patients with CKD increase their susceptibility to AKI. When AKI develops in this context, renal tubular epithelial cells frequently undergo maladaptive repair, contributing to ongoing renal function decline and accelerating progression to end-stage renal disease. Despite its clinical significance, the pathophysiological mechanisms and specific biomarkers associated with AoCKI remain insufficiently understood. Traditional serum creatinine measurements are limited by delayed response and lack of sensitivity. Recently, novel biomarkers such as TIMP-2, IGFBP7, NGAL, KIM-1, FGF-23, and PENK have shown preliminary validation in AoCKI populations, indicating substantial potential for clinical application (Table 2). Given the complexity of AoCKI, an optimal biomarker should reflect both the extent of current injury and the likelihood of impaired renal repair. The clinical course of AoCKI is often prolonged and characterized by heterogeneous manifestations, complicating prognosis based on single-time-point biomarker assessments. Nevertheless, research into AoCKI biomarkers remains clinically important. Early identification of high-risk patients enables targeted interventions; for instance, evaluating renal repair capacity through biomarkers before exposure to infection, surgery, or nephrotoxic agents can inform preventive strategies. Additionally, dynamic monitoring of biomarker trends may offer a more accurate assessment of renal recovery than isolated measurements, thereby supporting clinical decision-making. The identification of novel biomarkers may also reveal new therapeutic targets for AKI. Approaches such as KIM-1-targeted degradation, p53 inhibition, and microvascular protective interventions exhibit promising translational potential. As a prevalent yet under-investigated syndrome, AoCKI presents significant challenges in clarifying its poor prognosis and advancing biomarker research. Future studies should focus on refining research methodologies to enable early detection, precise risk stratification, and effective intervention, ultimately improving long-term outcomes for this high-risk patient population.

**TABLE 2:** Comparison of representative biomarkers for acute-on-chronic kidney injury.

Biomarker	Biological rationale	Diagnostic performance	Specificity for AoCKI	Clinical utility	Advantages	Limitations	Clinical validation
NGAL	Tubular epithelial injury	High sensitivity; early increase after AKI	Low–moderate (also elevated in CKD, sepsis and systemic inflammation)	Early diagnosis; risk stratification	Rapid response; extensively validated	Limited specificity in inflammatory conditions	Clinical
KIM-1	Proximal tubular injury	Moderate–high diagnostic accuracy	Moderate (kidney-specific but influenced by chronic tubular injury)	Diagnosis and prognosis	High renal specificity	Delayed elevation compared with NGAL	Clinical
TIMP-2 × IGFBP7	Cell-cycle arrest (G1 arrest)	Excellent predictive value for AKI risk	Moderate (reflects cellular stress rather than AoCKI itself)	Early risk prediction; ICU monitoring	FDA-approved; predicts AKI before creatinine rise	High cost; limited availability	Clinical
Cystatin C	Glomerular filtration marker	Moderate sensitivity; detects GFR decline earlier than creatinine	Low (not specific for AoCKI)	Renal function assessment	Less affected by muscle mass	Influenced by inflammation and thyroid dysfunction	Clinical
FGF23	Mineral metabolism dysregulation and CKD progression	Mainly prognostic	Low (reflects CKD severity rather than acute injury)	Prognostic assessment	Associated with CKD progression and cardiovascular risk	Limited value for early diagnosis	Emerging
Proenkephalin (PENK)	Functional decline in glomerular filtration	Promising early predictor of AKI	Moderate–high (less affected by muscle mass)	Early detection and prognosis	Reflects real-time renal function; stable biomarker	Limited AoCKI-specific validation	Emerging
